# Tea Polyphenols Mitigate TBBPA-Induced Renal Injury Through Modulation of ROS-PI3K/AKT-NF-κB Signalling in Carp (*Cyprinus carpio*)

**DOI:** 10.3390/ani15152307

**Published:** 2025-08-06

**Authors:** Fuxin Han, Ran Xu, Hongru Wang, Xuejiao Gao, Mengyao Guo

**Affiliations:** Department of Clinical Veterinary Medicine, College of Veterinary Medicine, Northeast Agricultural University, Harbin 150030, China; s230601062@neau.edu.cn (F.H.); xr1998_lucky@163.com (R.X.); s230601054@neau.edu.cn (H.W.)

**Keywords:** tea polyphenols, aquaculture feed additives, inflammatory injury, oxidative stress

## Abstract

Tetrabromobisphenol A (TBBPA), a widely utilised brominated flame retardant, demonstrates toxicological effects in aquatic organisms. Tea polyphenols (TPs), natural compounds found in tea leaves, exhibit both antioxidant and anti-inflammatory activities. The kidney is one of the major metabolic organs in common carp and serves as a target organ for toxic substances. This study evaluated the therapeutic potential of TPs in mitigating TBBPA-induced nephrotoxicity in common carp. Common carp were exposed to 0.5 mg/L TBBPA in water and/or fed a diet supplemented with 1 g/kg TPs for 14 days. In vitro, primary renal cells were treated with 60 μM TBBPA and/or 2.5 μg/L TPs for 24 h. Methods included histopathology, TUNEL assay for apoptosis, ROS detection, and molecular analyses. Antioxidant enzymes (SOD, CAT) and inflammatory cytokines (IL-1β, IL-6, TNF-α) were quantified using ELISA kits. Results showed that TBBPA induced oxidative stress, and activated the ROS-PI3K/AKT-NF-κB pathway, thereby resulting in inflammatory responses. TBBPA upregulated apoptosis-related genes (*Caspase-3*, *Bax*, and *Bcl-2*) and induced apoptosis. TBBPA upregulated the expression of RIPK3/MLKL, thereby exacerbating necroptosis. TPs intervention significantly mitigated these effects by reducing ROS, suppressing NF-κB activation, and restoring antioxidant enzyme activities (SOD, CAT). Moreover, TPs attenuated apoptosis and necrosis in the carp kidney, thereby enhancing the survival ability and immunity of common carp.

## Simple Summary

As a food-derived bioactive compound, TPs have garnered extensive attention for their physiological properties in research. In this study, we found that TPs lower ROS levels, restore antioxidant enzymes (SOD, CAT), and inhibit the ROS-PI3K/AKT-NF-κB pathway, preventing inflammatory injury. TPs also suppress apoptosis by regulating Caspase-3, Bax, and Bcl-2, and relieve necrosis by downregulating the RIPK3/MLKL signalling pathway. These findings suggest that dietary TPs may serve as a therapeutic agent against TBBPA-induced renal injury in common carp.

## 1. Introduction

Common carp (*Cyprinus carpio* L.) ranks as the third most cultivated freshwater fish globally. More than 90% of the global output comes from Asia, and it is one of the freshwater fish commonly consumed by Chinese people [1]. Common carp provides humans with quality animal protein and essential minerals, including iron and zinc. The renal system in carp plays a crucial role in the excretion of metabolic waste. Its function often makes it a target for toxic substances that cause tissue damage [2]. Kidney damage in carp prolongs the breeding cycle and even increases the mortality rate, resulting in greater difficulty in feeding and management [3].

Tetrabromobisphenol A (TBBPA), a widely used brominated flame retardant, is incorporated as an additive in plastics, textiles, and electronic equipment [4,5]. Due to its lipophilicity and thermal stability, TBBPA and its derivatives exhibit significant bioaccumulation and biomagnification in organisms, resulting in developmental, reproductive, and endocrine disruptions, particularly in rodent models and aquatic organisms [6,7,8]. Environmental monitoring has identified TBBPA at varying concentrations in air, sediments, and marine systems worldwide [9]. Asia accounts for approximately 80% of global TBBPA consumption, with China being the dominant consumer [10,11]. Aquatic sediments serve as a reservoir for TBBPA, and its toxic effects on marine organisms have been widely documented [12]. Yang et al. documented TBBPA concentrations as high as 4.87 μg/L in Chaohu Lake, representing the highest reported aqueous environmental concentration worldwide, particularly in grass carp (*Ctenopharyngodon idella*) and rodent models [13]. TBBPA bioaccumulation was detected in grass carp tissues from Chaohu Lake, with the highest concentrations observed in the kidney (75.2–162.4 ng/g dw), followed by the liver and muscle. TBBPA can activate the ROS/NF-κB pathway, leading to intestinal inflammation and cellular necrosis in common carp [14]. In vitro studies using grass carp hepatocytes have demonstrated that TBBPA induces apoptosis by elevating reactive oxygen species (ROS) levels and impairing ER-mitochondrial communication [15]. Fukuda et al. observed that neonatal rats orally administered 200 mg/kg and 600 mg/kg of TBBPA for 18 days developed multicystic kidney lesions, confirming its nephrotoxicity [16]. TBBPA-induced nephrotoxicity in rodents is primarily characterised by renal tubular abnormalities and cysts, as well as oxidative stress-mediated tissue injury, with juvenile individuals exhibiting higher susceptibility. A high dosage (typically ≥250 mg/kg bw) is a critical factor in triggering renal toxicity [16,17,18]. However, the mechanisms underlying TBBPA-induced renal injury in teleost fish remain to be elucidated.

The PI3K/AKT/NF-κB pathway plays a dual role in teleost kidney physiology, regulating immune responses and tissue repair while also contributing to renal injury and fibrosis when dysregulated. Paraquat exposure leads to apoptosis and programmed necrosis through oxidative stress and the PTEN/PI3K/AKT pathway, thereby causing immune dysfunction in CIK cells [19]. Albicanol antagonises hepatocyte apoptosis induced by Profenofos exposure through a ROS-mediated PTEN/PI3K/AKT pathway [20]. Tea polyphenols (TPs), the most abundant and biologically active natural compounds in Camellia sinensis leaves, exhibit significant physiological properties including antioxidant, anti-inflammatory, and immunomodulatory activities [21]. As food-derived bioactive compounds, TPs have garnered considerable attention in the development of functional feeds. Dietary supplementation with TPs has been shown to mitigate oxidative stress in the gills of common carp, reduce inflammatory responses, and enhance innate immune function [22]. Zhao et al. demonstrated that TPs attenuate ethionine-induced oxidative stress, inflammatory response, and apoptosis in Ctenopharyngodon idella kidney (CIK) cells by suppressing the ROS/MAPK/NF-κB pathway [23]. However, few studies have investigated the therapeutic effects of TPs on renal injury in carp, leaving it unclear if TPs can reduce TBBPA-induced renal injury. We established a TBBPA exposure model using common carp and primary renal cells to explore the mechanism of TBBPA-induced renal injury and the therapeutic efficacy of TPs, using histopathological examination and molecular biology techniques. This study offers novel insights into the treatment of TBBPA-induced renal injury in common carp.

## 2. Materials and Methods

### 2.1. Animals and Treatment

All experimental procedures involving animals were approved by the Institutional Animal Care and Use Committee of Northeast Agricultural University. Eighty healthy carp weighing 76.0 ± 5.4 g were obtained for this study from a nearby aquaculture facility. Each experimental group contained 20 fish (*n* = 20). The fish were randomly divided into four groups: Control Group (Con): fed basal diet only; TPs Group (TPs): fed basal diet supplemented with 1 g/kg TPs; TBBPA Group (TBBPA): exposed to 0.5 mg/L TBBPA in water while fed basal diet; TBBPA + TPs Group (TBBPA + TPs): fed basal diet with 1 g/kg TPs and exposed to 0.5 mg/L TBBPA in water. TPs were thoroughly mixed with the basal diet at 1 g/kg feed concentration. Fish were housed in the tanks with temperature controlled at 26 ± 2 °C and minimum dissolved oxygen levels above 7 mg/L. Throughout the 14-day experiment, fish were fed twice daily, with 30% water exchange performed every other day to maintain water quality. Subsequently, the common carp were anaesthetised with 0.02% MS-222 and euthanised. The kidneys were removed, a part of the kidneys rinsed with PBS, and fixed in 4% paraformaldehyde. The remaining kidney segments were frozen in liquid nitrogen and stored at −80 °C for later tests.

### 2.2. Culture and Treatment of Primary Renal Cells from Carp Kidney

Healthy carp were selected, then the body surface was first wiped with 0.1% potassium permanganate solution, followed by disinfection with 75% ethanol at the dissection site. Approximately 0.5 cm^3^ of renal tissue was aseptically excised. After removal of surface blood clots and peritoneal tissue, the tissue was minced and rinsed 3–4 times with phosphate-buffered saline (PBS). The renal tissues were transferred into EP tubes, digested with 0.25% trypsin, and placed in a 28 °C water bath for 5 min with manual shaking every minute. After digestion, the trypsin solution was aspirated, and the tissues were completely dissociated with complete MI99 medium [24]. The cells were then seeded into six-well plates at densities ranging from 2 × 10^6^ to 8 × 10^6^ cells per well. The culture medium consisted of complete MI99 medium supplemented with 12% fetal bovine serum (Haixing Biological Technology Co., Ltd., Suzhou, China) and 1% penicillin-streptomycin (Solarbio Science & Technology Co., Ltd., Beijing, China). Cells were maintained at 28 °C in a 5% CO_2_ incubator. When the cell reached 80% confluency, the cultures were divided into three groups: Control group, 60 μM TBBPA group, 2.5 μg/L TPs group, and a 60 μM TBBPA + 2.5 μg/L TPs group. After 24 h of treatment, cells were harvested for subsequent analysis.

Cell viability was assessed using a Cell Counting Kit-8 (CCK-8; Jiancheng Biotechnology Co., Ltd., Nanjing, China). Renal primary cells were seeded in 96-well plates, and complete medium supplemented with varying concentrations of TBBPA (0, 20, 40, 60, 80, 100, or 120 μM) was added to each well. The Con group received medium with 0.1% DMSO (*v*/*v*). After 48 h of incubation, the medium was discarded, and 10 μL of CCK-8 solution was added to each well before incubation at 28 °C for 2 h. The absorbance was measured at 450 nm using a microplate reader. The viability of primary renal cells could reach 80% after being treated with 60 μM TBBPA (Figure S1). Then, 60 μM TBBPA was used for subsequent experiments.

### 2.3. Histological Analysis

Fresh carp renal tissues were fixed in 4% paraformaldehyde (PFA), dehydrated through a graded ethanol series, cleared in xylene, and embedded in liquid paraffin. After solidification, the paraffin blocks were sectioned at 5 μm thickness. Following dewaxing and hydration, the sections were stained with hematoxylin and eosin (HE) [25]. The stained sections were sealed, and the morphology of the renal tissues was observed and photographed under a microscope (Olympus Optical Co., Ltd., Tokyo, Japan). Three tissue blocks per group were analysed (*n* = 3).

### 2.4. TUNEL Analysis

Renal tissues were rinsed with PBS before paraffin embedding. The paraffin-embedded sections were dewaxed in xylene and rehydrated through a graded ethanol series (70%, 80%, 95%, 100%), followed by incubation with proteinase K working solution at 37 °C. Subsequently, the sections were sequentially stained with terminal deoxynucleotidyl transferase (TdT) and deoxyuridine triphosphate (dUTP) solutions. Following reaction termination, the sections were washed to remove residual reagents and mounted for microscopic analysis [26]. Fluorescence microscopy (Olympus Optical Co., Ltd., Tokyo, Japan) was performed to examine the fluorescence signals in the renal tissues. Three tissue blocks per group were analysed (*n* = 3).

### 2.5. Oxygen Radical Detection

The renal tissue was trimmed into 1 mm^3^ pieces and sectioned. Using fine-tipped forceps, the tissue was gently dissociated by rubbing against a 300-mesh nylon net, followed by PBS rinsing until complete tissue dissociation was achieved. A single-cell suspension was prepared through resuspension, then incubated with a diluted DCFH-DA probe at 37 °C for 30 min. The probe-labeled single-cell suspension was collected, washed, and resuspended. Fluorescence intensity was measured using a microplate fluorometer at an excitation wavelength of 500 nm and an emission wavelength of 525 nm [22]. For the in vitro experiments, the supernatant from TBBPA- and TPs-treated cells was discarded, and cells were washed three times with PBS. The DCFH-DA probe was prepared by 1:1000 dilution in serum-free medium, and 1 mL of the diluted probe was added to each well. Following incubation at 37 °C for 30 min, cells were washed three times with PBS and analysed by fluorescence microscopy [27]. The ROS kit was purchased from Nanjing Jiancheng Biotechnology Co., Ltd., Nanjing, China.

### 2.6. AO/EB Staining

Necrosis was observed using the acridine orange/ethidium bromide (AO/EB) double dye kit (Leagene, Biotechnology Co., Ltd., Beijing, China). The treated cells were harvested and mixed with AO/EB (acridine orange/ethidium bromide) at a 1:1 ratio in PBS to prepare a working solution (10 μg/mL). The cell suspension was then replated in 6-well plates and visualised by fluorescence microscopy (Olympus Optical Co., Ltd., Tokyo, Japan) [28]. Each group included three independent biological replicates (*n* = 3). Fluorescence images were acquired. Quantitative analysis was performed using ImageJ 1.53e software.

### 2.7. Antioxidant Enzyme Detection

A 10% tissue homogenate was prepared by mixing 900 mL of physiological saline with 0.1 g of sample in a homogeniser maintained in an ice bath. Collect the primary renal cells, wash them 1–2 times with PBS, then centrifuge at low speed to pellet the cells. Resuspend the cell pellet in 0.3–0.5 mL of PBS buffer, and homogenise the cells by grinding. Centrifuge at 3500 rpm for 10 min, and collect the supernatant for the chromogenic reaction. Each group included three independent biological replicates (*n* = 3). Protein concentrations in tissues and cells were determined using a BCA protein assay kit (Thermo Fisher Scientific, Waltham, MA, USA). The levels of superoxide dismutase (SOD), glutathione (GSH), malondialdehyde (MDA), and catalase (CAT) in tissues and cells were quantified using corresponding assay kits (Jiancheng Biotechnology Co., Ltd., Nanjing, China) according to the manufacturer’s instructions.

### 2.8. Enzyme-Linked Immunosorbent Assay (ELISA)

Protein expression levels of interleukin-1β (IL-1β), interleukin-6 (IL-6), and tumour necrosis factor-α (TNF-α) were measured in both renal tissues and isolated primary renal cells using commercial ELISA kits from Andy Gene Biotechnology, China (catalogue numbers: E-43902 for IL-1β, E-53219 for IL-6, and E-43904 for TNF-α). Each experimental group included three biological replicates. All assay procedures, including sample preparation and incubation conditions, were strictly followed according to the manufacturer’s instructions. The absorbance was measured at 450 nm using a Multiskan SkyHigh microplate reader (Thermo Fisher Scientific, Waltham, MA, USA).

### 2.9. Real-Time PCR

Renal tissues and primary renal cells were lysed with Trizol reagent (Thermo Fisher Scientific, Waltham, MA, USA) following the manufacturer’s protocol for RNA extraction. Total RNA concentration was assessed by UV spectrophotometry (Thermo Fisher Scientific, Waltham, MA, USA). RNA was reverse transcribed into complementary DNA (cDNA) using a reverse transcription kit (Vazyme Biotech Co., Ltd., Nanjing, China). Target genes were denatured, annealed, and amplified in a 20 μL reaction system using a PCR 2720 Thermal Cycler (Thermo Fisher Scientific, Waltham, MA, USA) and an ABI 7500 Real-Time PCR System. Glyceraldehyde-3-phosphate dehydrogenase (GAPDH) was used as the internal reference gene, and the results were expressed as 2^−ΔΔCt^. Each group included three independent biological replicates (*n* = 3). Primer sequences are listed in Table 1.

### 2.10. Western Blotting Assay

Proteins were extracted from the kidney and renal progenitor cells using lysis buffer, the optical density (OD) values of standards and samples were measured using a BCA Protein Assay Kit (Jiancheng Biotechnology Co., Ltd., Nanjing, China), and standard curves were generated to calculate sample protein concentrations. An appropriate SDS-polyacrylamide gel concentration was selected based on the molecular weight of the target proteins. The target bands were cut out according to the molecular weight markers. Target protein bands were excised according to molecular weight markers and transferred to nitrocellulose membranes by wet blotting at 220 mA. After transfer, the membrane was blocked with 5% skim milk for 2 h at room temperature, then incubated with primary antibody at 4 °C overnight. Subsequently, the membrane was incubated with diluted secondary antibodies (goat anti-rabbit IgG and goat anti-mouse IgG) for 2 h at room temperature in the dark for signal detection [29]. Each group included three independent biological replicates (*n* = 3). Antibodies used for Western blot analysis are listed in Table 2.

### 2.11. Statistical Analysis

Data were analysed using GraphPad Prism 10 software. One-way analysis of variance (ANOVA) was used to calculate the difference between the values. All experiments included triplicate biological replicates (*n* = 3). Statistical significance was determined as follows: (*, *p* < 0.05; **, *p* < 0.01; ***, *p* < 0.001; ****, *p* < 0.0001).

## 3. Results

### 3.1. TPs Mitigated TBBPA-Induced Renal Injury

**Figure 1:**
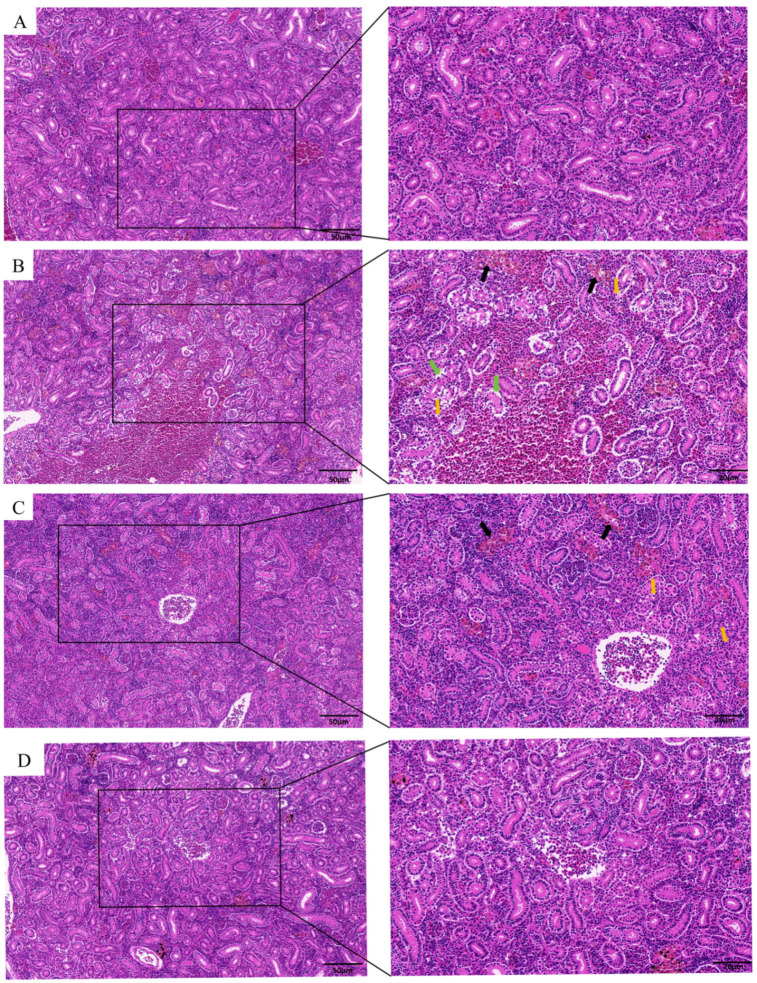
**Effects of TBBPA exposure and TPs intervention on the morphology of carp kidney.** (**A**) HE staining of kidney tissue in the Con group. (**B**) HE staining of kidney tissue in the TBBPA group. (**C**) HE staining of kidney tissue in the TBBPA + TPs group. (**D**) HE staining of kidney tissue in the TPs group. The black arrows in the figure indicate congestion of the renal tissue, the yellow arrows indicate inflammatory cell infiltration, and the green arrows indicate oedema of the renal tubules. The left figure is magnified 20×, and the right figure is magnified 40×. Each group included three independent biological replicates (*n* = 3). The data are expressed as mean ± SEM and analysed using one-way analysis of variance (ANOVA).

Histopathological analysis demonstrated that TBBPA exposure induced renal damage compared to the Con group (Figure 1A,B), characterised by renal tubular oedema and rupture, tissue congestion, and inflammatory cell infiltration. After TPs intervention, although residual congestion persisted in renal tissues, renal tubular morphology showed improvement (Figure 1C). No morphological alterations were observed in the TPs group (Figure 1D).

### 3.2. TPs Mitigated the Oxidative Stress Induced by TBBPA in Renal Tissue

**Figure 2:**
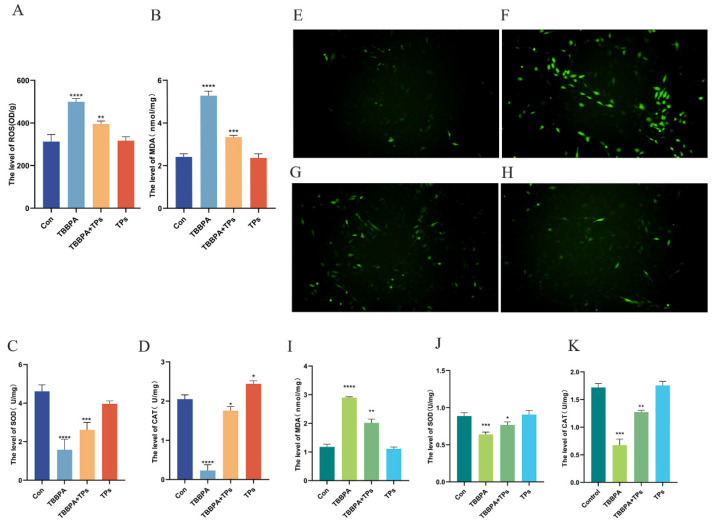
**Effects of TBBPA exposure and TPs intervention on renal oxidative stress of common carp.** (**A**) Changes in ROS levels in the kidney. (**B**) MDA content was detected in kidney tissue. (**C**) SOD content was detected in the kidney. (**D**) CAT content was detected in the kidney. (**E**–**H**) Changes in the content of ROS in cells, magnification 10×. (**I**) Detection of MDA content in primary renal cells. (**J**) Detection of SOD content in primary renal cells. (**K**) Detection of CAT content in primary renal cells. Each group consisted of three independent biological replicates (*n* = 3). Data are expressed as mean ± SEM. Analysis was performed using one-way ANOVA. *, *p* < 0.05; **, *p* < 0.01; ***, *p* < 0.001; ****, *p* < 0.0001.

Compared to the Con group, the TBBPA group exhibited significantly higher ROS levels in carp renal tissues (*p* < 0.0001). TPs treatment reduced ROS accumulation (Figure 2A). Fluorescence was used to detect the expression of reactive oxygen species (ROS) in primary renal cells from carp kidney. TBBPA exposure elevated oxidative stress levels in cells. The green fluorescence intensity was reduced by TPs treatment (Figure 2E–H). The results showed that MDA levels were significantly higher (*p* < 0.0001) in the TBBPA group than in the Con group in tissue and cells, but TPs treatment effectively reduced them (*p* < 0.001) (Figure 2B,I). Antioxidant enzyme activity results are presented in Figure 2C,D,J,K. Compared with the Con group, the TBBPA group exhibited reduced CAT and SOD levels, whereas TPs treatment significantly enhanced (*p* < 0.001) their activities.

### 3.3. TPs Mitigated TBBPA-Induced Renal Inflammatory Injury

**Figure 3:**
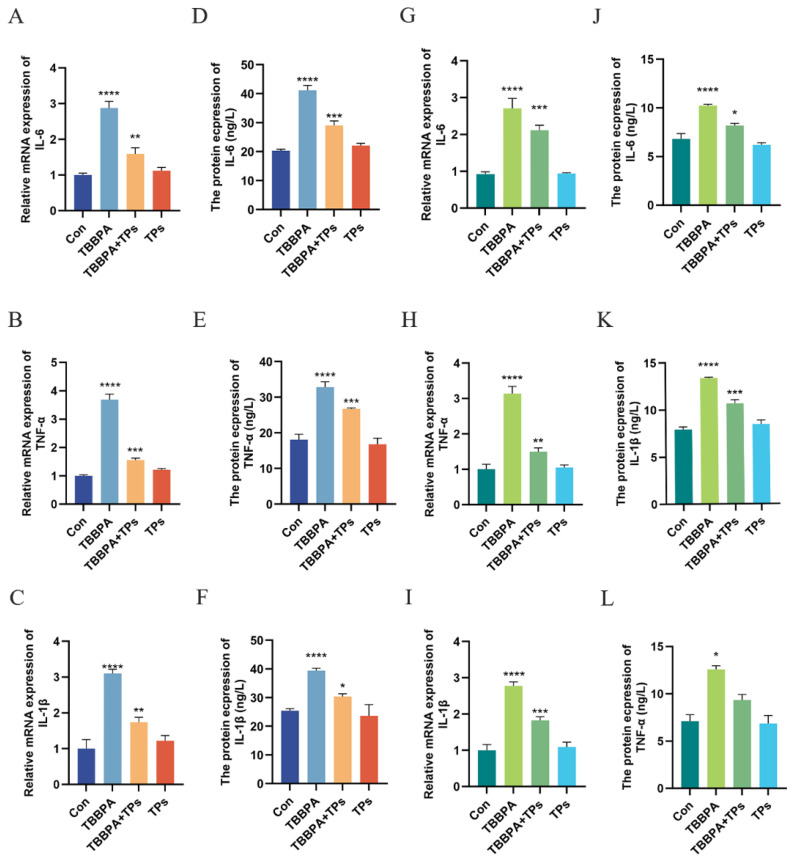
**Effects of TBBPA exposure and TPs intervention on the levels of inflammatory factors in renal tissue.** (**A**–**C**) The mRNA levels of inflammatory factors in renal tissue. (**D**–**F**) Protein levels of inflammatory factors in renal tissue. (**G**–**I**) Expression levels of mRNA of inflammatory factors in primary renal cells. (**J**–**L**) Expression levels of the protein of inflammatory factors in primary renal cells. Each group included three independent biological replicates (*n* = 3). The data are expressed as mean ± SEM. Analysis was performed using one-way ANOVA. *, *p* < 0.05; **, *p* < 0.01; ***, *p* < 0.001; ****, *p* < 0.0001.

To further evaluate renal inflammatory responses, we examined the expression of inflammatory factors IL-1β, IL-6, and TNF-α in both tissue and cells. The results demonstrated that the mRNA levels of *IL-1β*, *IL-6*, and *TNF-α* remained comparable to those of the Con group when TPs were given alone, whereas the mRNA levels of *IL-1β*, *IL-6*, and *TNF-α* were significantly elevated (*p* < 0.0001) by TBBPA exposure (Figure 3A–C,G–I). As shown in Figure 3D–L, the protein levels of inflammatory factors followed the same trend as their mRNA counterparts. The levels of inflammatory factors were significantly increased (*p* < 0.0001) in the TBBPA group. The levels of inflammatory factors were decreased after the TPs intervention.

### 3.4. TPs Mitigated TBBPA-Induced Activation of the PI3K/AKT/NF-κB Pathway

**Figure 4:**
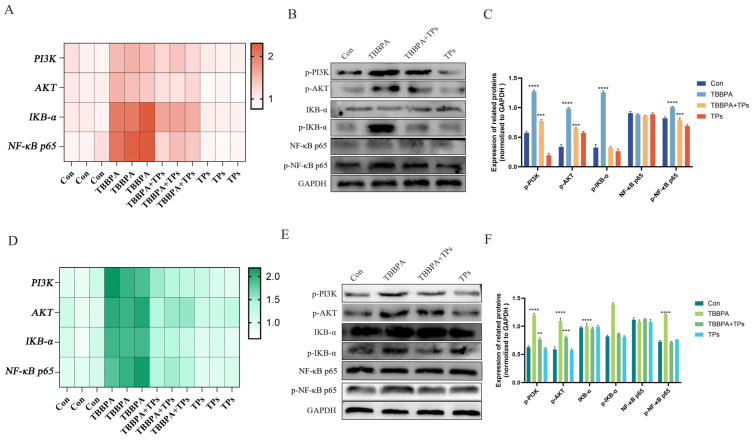
**Effects of TBBPA exposure and TPs intervention on the expression of the PI3K/AKT-NF-κB pathway.** (**A**) The mRNA levels of key proteins in the PI3K/AKT/NF-κB pathway in renal tissue. (**B**,**C**) Expression levels of key proteins in the PI3K/AKT/NF-κB pathway in renal tissue. (**D**) The mRNA levels of key proteins in the PI3K/AKT/NF-κB pathway in primary renal cells. (**E**,**F**) Levels of key proteins in the PI3K/AKT/NF-κB pathway in primary renal cells. Each group included three independent biological replicates (*n* = 3). Data are expressed as mean ± SEM. Analysis was performed using one-way analysis of variance (ANOVA). **, *p* < 0.01; ***, *p* < 0.001; ****, *p* < 0.0001.

As shown in Figure 4A–F, TBBPA exposure significantly increased (*p* < 0.0001) both PI3K and AKT levels at the mRNA and protein levels in tissues and cells compared with the Con group. p-PI3K and p-AKT were also markedly increased (*p* < 0.0001) in the TBBPA group. TPs treatment significantly reduced (*p* < 0.0001) these phosphorylation levels. TBBPA exposure significantly upregulated (*p* < 0.0001) phosphorylated IKB-α (p-IKB-α) and phosphorylated NF-κB (p-NF-κB) at both the transcriptional and translational levels (Figure 4A–F) compared to the Con group. TPs treatment significantly decreased (*p* < 0.001) the protein levels of both p-IKB-α and p-NF-κB.

### 3.5. TPs Reduced TBBPA-Induced Renal Cell Apoptosis

**Figure 5:**
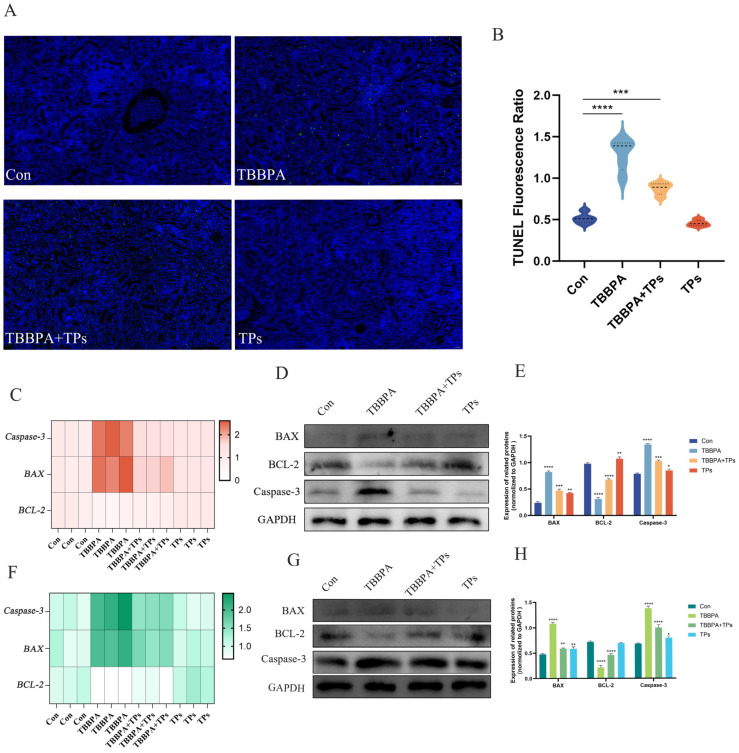
**Effects of TBBPA exposure and TPs intervention on renal apoptosis of common carp.** (**A**) TUNEL results of kidney tissue; green represents apoptosis. (**B**) Tunnel fluorescence ratio. (**C**) The mRNA expression of *Caspase-3*, *BAX*, and *BCL-2* genes in the kidney. (**D**,**E**) Protein expression levels of Caspase-3, BAX, and BCL-2 in the kidney. (**F**) The mRNA expression of Caspase-3, BAX, and BCL-2 in primary renal cells. (**G**,**H**) Protein expression of the *Caspase-3* gene and *BAX* and *BCL-2* in primary renal cells. Each group included three independent biological replicates (*n* = 3). Data are expressed as mean ± SEM. Analysis was performed using one-way ANOVA. *, *p* < 0.05; **, *p* < 0.01; ***, *p* < 0.001; ****, *p* < 0.0001.

TUNEL results demonstrated that the number of apoptotic cells in kidney tissues was significantly elevated (*p <* 0.001) in the TBBPA group compared with the Con group. This apoptotic effect was significantly reduced (*p* < 0.0001) after TPs intervention (Figure 5A,B). As shown in Figure 5C,F, the mRNA levels of *Caspase-3* and *BAX* were significantly increased (*p* < 0.001) in the renal tissue and primary renal cells after TBBPA exposure, while the mRNA levels of the anti-apoptotic gene *BCL-2* were significantly decreased (*p* < 0.0001). TPs intervention decreased (*p* < 0.05) *Caspase-3* and *BAX* mRNA levels while increasing *BCL-2* expression. Western blot results similarly showed that the protein levels of Caspase-3 and BAX were significantly increased (*p* < 0.0001) after TBBPA exposure (Figure 5D,E,G,H). At the same time, those of BCL-2 were significantly decreased (*p* < 0.0001). Caspase-3 and BAX protein levels were significantly decreased (*p* < 0.001), downregulated, and BCL-2 protein levels were significantly increased, upregulated, after TPs intervention (Figure 5D,E,G,H).

### 3.6. TPs Mitigated TBBPA-Induced Renal Cell Necrosis

**Figure 6:**
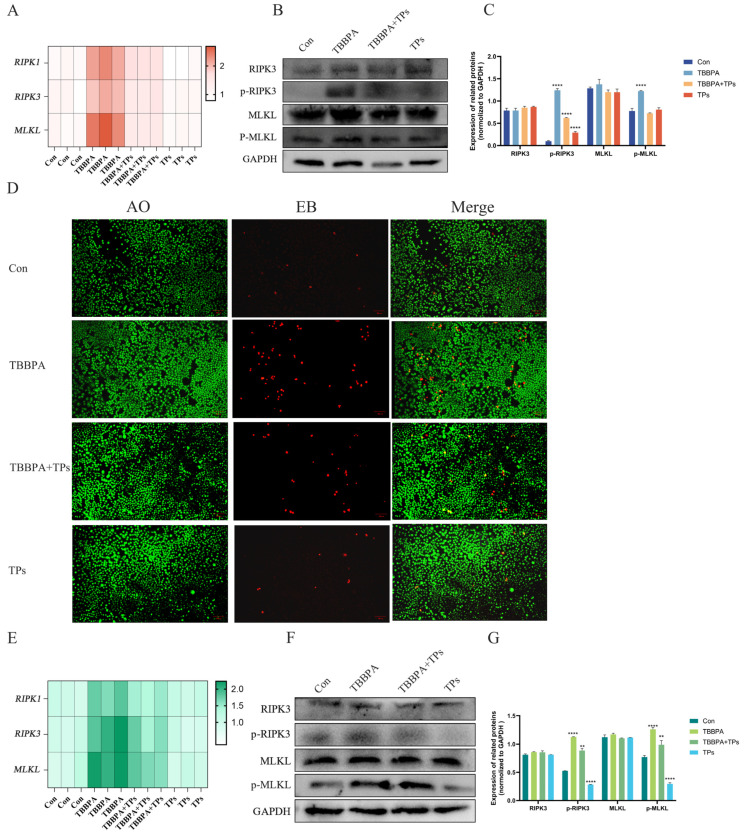
**Effects of TBBPA exposure and TPs intervention on renal necrosis of carp.** (**A**) The mRNA expression of *RIPK1*, *RIPK3*, and *MLKL* genes in the kidney. (**B**,**C**) Expression levels of RIPK1, RIPK3, and MLKL proteins in the kidney. (**D**) AO/EB results. Orange represents apoptotic cells, and red represents necrotic cells. Magnification 10×. (**E**) The mRNA expression of *RIPK1*, *RIPK3*, and *MLKL* genes in renal primary cells. (**F**,**G**) Protein expression levels of RIPK1, RIPK3, and MLKL in primary renal cells. Each group included three independent biological replicates (*n* = 3). Data are expressed as mean ± SEM. Analysis was performed using one-way analysis of variance (ANOVA). **, *p* < 0.01; ****, *p* < 0.0001.

Figure 6A,E demonstrate that TBBPA exposure significantly elevated (*p* < 0.001) the mRNA levels of receptor-interacting protein kinase 1 (*RIPK1*), receptor-interacting protein kinase 3 (*RIPK3*), and mixed lineage kinase domain-like protein (*MLKL*) in tissues and cells, while TPs intervention effectively reversed this effect. The results of acridine orange and ethidium bromide (AO/EB) staining are shown in Figure 6D. Compared with the Con group, the number of apoptotic and necrotic cells after TBBPA exposure was increased, and the number of apoptotic and necrotic cells after TPs intervention was significantly decreased. Western blot analysis revealed that the protein levels of phosphorylated RIPK3 (p-RIPK3) and phosphorylated MLKL (p-MLKL) were markedly upregulated (*p* < 0.0001) upon TBBPA treatment, which were subsequently attenuated by TPs administration (Figure 6B,C,F,G).

## 4. Discussion

TBBPA is the most widely produced brominated flame retardant globally and is a widespread persistent organic pollutant in the environment [5]. Studies have demonstrated that TBBPA adversely affects the survival, reproduction, and development of various aquatic organisms [30]. Tea polyphenols are a general term for polyphenols in tea, which exhibit pharmacological effects such as antioxidant, antiviral, and antibacterial properties [31]. Tea polyphenols (TPs) not only enhance the antioxidant capacity of common carp but also modulate the gut microbiota composition, thereby preventing intestinal barrier dysfunction. Specifically, TPs alleviate *Aeromonas hydrophila*-induced intestinal barrier damage in grass carp by inhibiting the RhoA/ROCK signalling pathway, and upregulating the expression of tight junction and adherens junction proteins [32]. Additionally, TPs enhance non-specific immunity and exert anti-inflammatory effects in koi carp by suppressing pro-inflammatory cytokines (IL-1β, IL-6) via the NF-κB pathway, while altering gut microbial structure (increasing Proteobacteria and reducing Fusobacteria abundance) [33]. The aim of this study was to investigate the protective effects of tea polyphenols on TBBPA-induced renal inflammation, apoptosis, and necroptosis. The results indicated that TBBPA induced inflammation, apoptosis, and necroptosis in kidney tissues and primary renal cells of common carp, whereas TPs intervention mitigated these effects.

The common carp is a freshwater fish of the genus Cyprinus. The kidneys of freshwater fish play a pivotal role in maintaining water and electrolyte homeostasis. In freshwater environments, fish excrete excess water while retaining salts via renal filtration and active tubular reabsorption, particularly of sodium chloride, to adapt to hypoosmotic conditions [34,35]. This process relies on efficient glomerular filtration and tubular reabsorption mechanisms. At the molecular level, TBBPA may interfere with renal excretory function by reacting with biotransformation enzymes such as glutathione, reducing its bioavailability [36]. Freshwater fish kidneys regulate acid-base balance and ammonia excretion, primarily through NH_4_^+^ elimination and H^+^/HCO_3_^−^ reabsorption. However, ammonia imbalance induces renal oxidative stress and structural damage. Zhang et al. [37] demonstrated that ammonia exposure upregulates CPS I expression in yellow catfish kidneys, activating glutamine synthesis while still causing oxidative stress and immunosuppression. Dietary supplementation with 5% TCE (Thalassodendron ciliatum extract) enhances antioxidant capacity in tilapia, mitigates ammonia-induced oxidative stress, and may provide renal protection [38].

An imbalance between oxidation and antioxidant defence systems leads to excessive ROS production. This triggers oxidative stress, which ultimately causes tissue damage. Zhang et al. demonstrated that TBBPA induces ROS overproduction and upregulates antioxidant enzymes. Malondialdehyde (MDA), an oxidative stress marker, induced oxidative stress in L02 cells [39]. The results showed that elevated concentrations of ROS and MDA were observed in kidney cells after TBBPA exposure. SOD acts as the primary defence against ROS by detoxifying superoxide anions. CAT, on the other hand, scavenges hydrogen peroxide, which further protects the cells from the damage caused by ROS [40]. TBBPA intervention significantly reduces the expression of SOD and CAT in mouse skeletal muscle and C2C12 cells [41]. Consistently, this study showed that the expression levels of SOD and CAT were significantly decreased in the kidney and primary renal cells of common carp after TBBPA treatment. TPs are potent antioxidants that scavenge free radicals and regulate cellular ROS levels, counteracting oxidative damage [42]. Studies have demonstrated that TPs increase serum CAT and SOD while decreasing MDA production in rats [43]. These findings indicate that tea polyphenols can alleviate oxidative stress and enhance endogenous antioxidant defences. The results of this study demonstrate that dietary TPs can scavenge excess ROS and reduce MDA levels, thereby enhancing the antioxidant capacity in the kidneys of common carp. The present findings are in concordance with the well-documented antioxidant properties of TPs reported in prior studies.

It has been shown that ROS are important regulatory molecules of the PI3K/AKT pathway and have a direct effect on AKT under oxidative stress [44]. The PI3K/AKT pathway serves as an important upstream regulator of the NF-κB signalling pathway [45]. PI3K activation converts phosphatidylinositol 4,5-bisphosphate (PIP2) to phosphatidylinositol 3,4,5-trisphosphate (PIP3), subsequently activating AKT. This activation leads to the release of inhibited NF-κB by phosphorylating IKB-α, which in turn initiates the transcription of pro-inflammatory genes [46]. In this study, the PI3K/AKT/NF-κB pathway was activated, and the expression levels of IL-1β, IL-6, and TNF-α were increased in common carp renal tissue and primary renal cells after TBBPA exposure. These results demonstrate that TBBPA activates the PI3K/AKT/NF-κB signalling pathway, thereby upregulating inflammatory factors and triggering inflammatory responses. TPs are considered natural compounds with anti-inflammatory potential. Previous studies have shown that green tea polyphenols can mitigate TBBPA-induced oxidative stress and alleviate inflammatory responses by inhibiting NF-κB activation and reducing lung injury in mice [47]. The current study demonstrated that TPs inhibited PI3K/AKT/NF-κB pathway activation and reduced production of inflammatory factors (IL-1β, IL-6, and TNF-α).

The pro-apoptotic proteins Caspase-3 and Bax and the anti-apoptotic protein Bcl-2 can regulate apoptosis. Xu et al. demonstrated that TBBPA induces gastric mucosal apoptosis by upregulating Caspase-3 through ROS-mediated NF-κB activation [8]. This study demonstrated that TBBPA exposure upregulated Caspase-3 and BAX while downregulating BCL-2 in common carp renal tissues and primary renal cells, indicating TBBPA activates apoptotic pathways through these molecular changes. Ma et al. demonstrated that against TBBPA-induced damage, TPs could enhance the antioxidant capacity and decrease the expression of pro-apoptotic genes in the gills of grass carp, thereby inhibiting excessive apoptosis and alleviating inflammation [33]. Consistent with these findings, our study demonstrated that TPs protected common carp renal tissues by attenuating apoptosis. TPs also reduce apoptosis-related genes (*Caspase-3*, *BAX*, *BCL-2*) and attenuate apoptosis in renal tissues and primary renal cells.

Cell death is a crucial process in organism development and homeostasis, with apoptosis and necrosis being the two main modes of cell death [4]. RIPK3 and its substrate MLKL are considered core cellular regulators of programmed necrosis, while TNF family cytokines such as TNF-α can mediate activation of the RIPK3/MLKL pathway [48]. Xu et al. showed that TBBPA exposure significantly increased the expression levels of necroptosis-related genes RIPK3 and MLKL, leading to gastric mucosal necrosis in mice. The results of this study showed that the expression levels of necrosis-associated genes RIPK1, RIPK3, and MLKL were significantly elevated in common carp kidneys and renal progenitor cells after TBBPA exposure, indicating that TBBPA exposure activates the RIPK3/MLKL signalling pathway to induce necrosis in common carp kidneys.

## 5. Conclusions

Dietary supplementation with TPs suppresses TBBPA-induced excessive ROS production, thereby alleviating oxidative stress in the kidney and primary renal cells of common carp. This inhibition further blocks the PI3K/AKT/NF-κB signaling pathway, mitigating inflammatory injury, apoptosis, and necroptosis. The potential synergistic effects between tea polyphenols and other antioxidants (e.g., vitamin E, selenium) warrant further investigation.

## Figures and Tables

**Table 1:** The primers used in the present study.

Gene	Forward Primer (5′→3′)	Reverse Primer (5′→3′)	Accession Number
*PI3K*	CGGGAAACGAGCTCAATCAT	CTCTCCAACAAATCCGCTCG	KY763989
*AKT*	CAACGGATGCGTCGTCTTCA	GTGTGAGTCTCCAAACCCCT	JX307852
*IKB-α*	GGCTACGCCAAAGACCTG	CGGACCTCGCCATTCATA	AK313421
*NF-κB p65*	GAAGAAGGATGTGGGAGATG	TGTTGTCGTAGATGGGCTGAG	MN167531
*IL-1β*	CTTCCACCCTCACAAACACATTCAAC	AATATAGCGTCCAAGGCGTTCCATC	EU047716
*IL-6*	CCGCATGGACTCGCAAGACG	CGGTAGTTGATGTACTCGTCCTCC	KC858890
*TNF-α*	TCATGGGAGTAAGGCTGGTATT	TCTTCAAAGGAATACAGGGGCT	LN593053
*Caspase-3*	GCTGTGCTTCGTTAGTGT	GAACCAAGAACCGCTCAT	JAEOAB010000019
*Bax*	ATGCGTGAATAAGGAGATGA	AGACCGAAGACCGTTACT	KJ174685
*Bcl-2*	GATACCGCAAGATTCCATACCC	TCCTTTCTATCTCGTCTCCAG	KJ174686
*RIPK1*	GGCTGCGTCGTTTGATA	GTTGGCACCCACGTTCT	MN123251
*RIPK3*	CAACGATGCCGTCTATGA	GAAGGAGCTGTTTGGTGTCT	OY720469
*MLKL*	CTGGCACAACAATCTGA	GAGACGCTGTAGAAGGAC	BC028141
*GAPDH*	GTTACAAGGGAGAAGTTCACCAT	CCGGTAGACTCGACTACATACAG	AJ870982

**Table 2:** Antibodies required for Western blot.

Name	Cat No.	Company	Dilution Times
p-PI3K	AF5905	Beyotime Biotechnology Co., Ltd., Shanghai, China	1:2000
P-AKT	AA329	Beyotime Biotechnology Co., Ltd., Shanghai, China	1:1000
NF-κB p65	AF1234	Beyotime Biotechnology Co., Ltd., Shanghai, China	1:1000
P-NF-κB p65	AF5875	Beyotime Biotechnology Co., Ltd., Shanghai, China	1:1000
IKB-α	AF1282	Beyotime Biotechnology Co., Ltd., Shanghai, China	1:1000
p-IKB-α	AF1870	Beyotime Biotechnology Co., Ltd., Shanghai, China	1:1000
Bax	WL01637	Wanlei Life Sciences Co., Ltd., Shenyang, China	1:500
Bcl-2	WL01556	Wanlei Life Sciences Co., Ltd., Shenyang, China	1:500
Caspase-3	WL04004	Wanlei Life Sciences Co., Ltd., Shenyang, China	1:500
RIPK3	47928T	Cell Signaling Technology, Inc., Boston, MA, USA	1:1000
p-RIPK3	93654T	Cell Signaling Technology, Inc., Boston, MA, USA	1:1000
MLKL	14993T	Cell Signaling Technology, Inc., Boston, MA, USA	1:1000
p-MLKL	3733T	Cell Signaling Technology, Inc., Boston, MA, USA	1:1000
GAPDH	WL01114	Wanlei Life Sciences Co., Ltd., Shenyang, China	1:1000

## Data Availability

Data is contained within the article or Supplementary Material.

## Supplementary Materials

The following supporting information can be downloaded at: https://www.mdpi.com/article/10.3390/ani15152307/s1, Figure S1: Effects of TBBPA on the viability of primary renal cells. * *p* ≤ 0.05; ** *p* ≤ 0.01; *** *p* ≤ 0.001. *p <*  0.05 indicates significant differences.
