# Supplementary material for: Tea Polyphenols Mitigate TBBPA-Induced Renal Injury Through Modulation of ROS-PI3K/AKT-NF-κB Signalling in Carp (Cyprinus carpio)
Source: Animals (Basel). 2025 Aug 6;15(15):2307. doi: 10.3390/ani15152307 (PMC12345511; doi:10.3390/ani15152307)

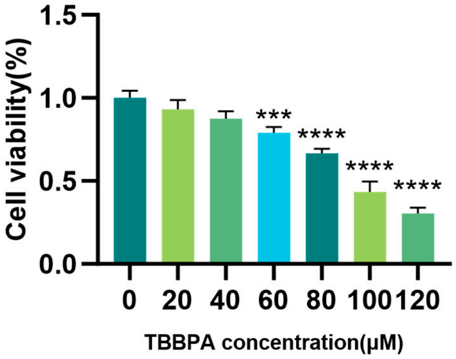


**Figure S1.** Effects of TBBPA on the viability of primary renal cells. *$P \leq 0.05$, **$P \leq 0.01$, ***$P \leq 0.001$. $P < 0.05$ indicates significant differences.

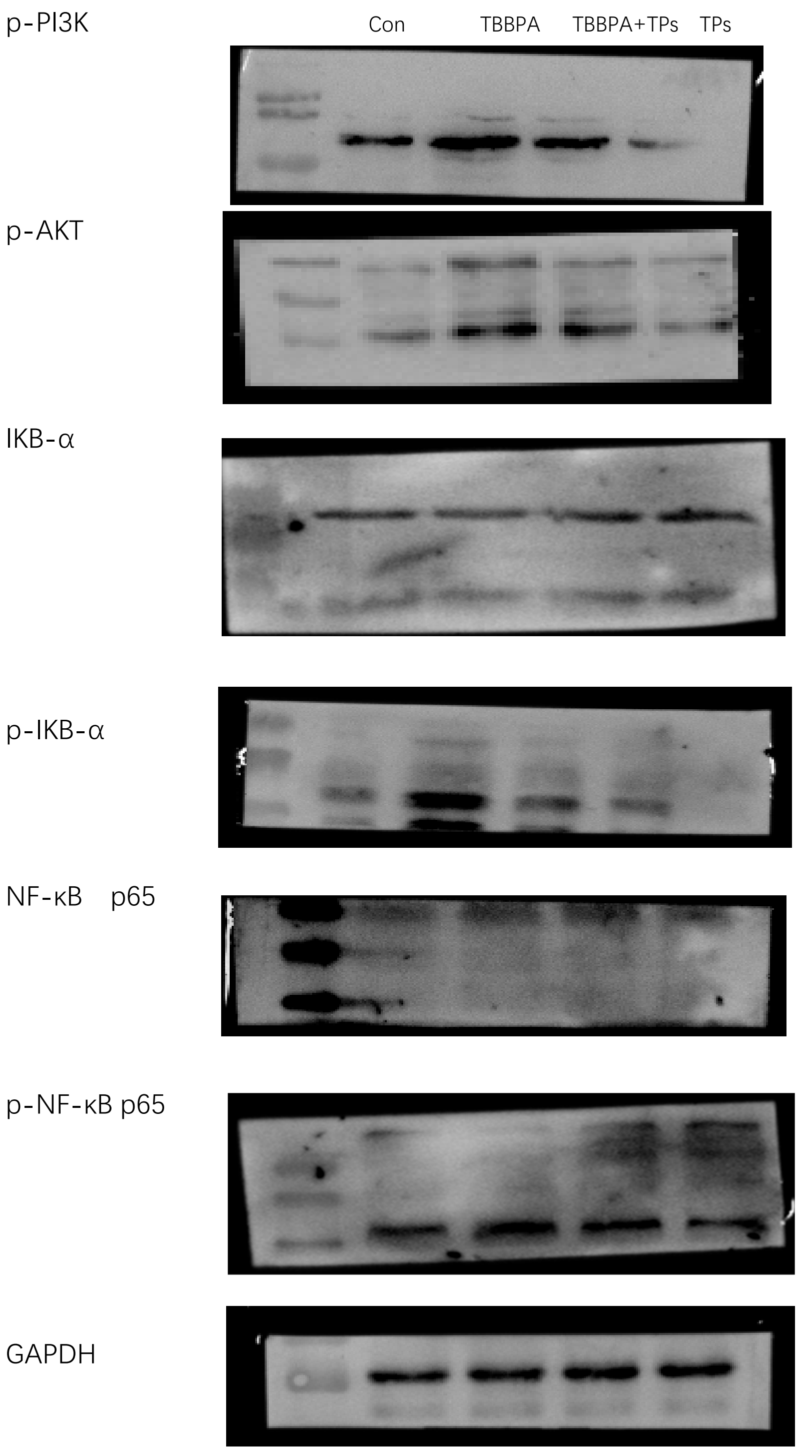
p-PI3K
Con
TBBPA
TBBPA+TPs
TPs
p-AKT
IKB-α
p-IKB-α
NF-κB p65
p-NF-κB p65
GAPDH

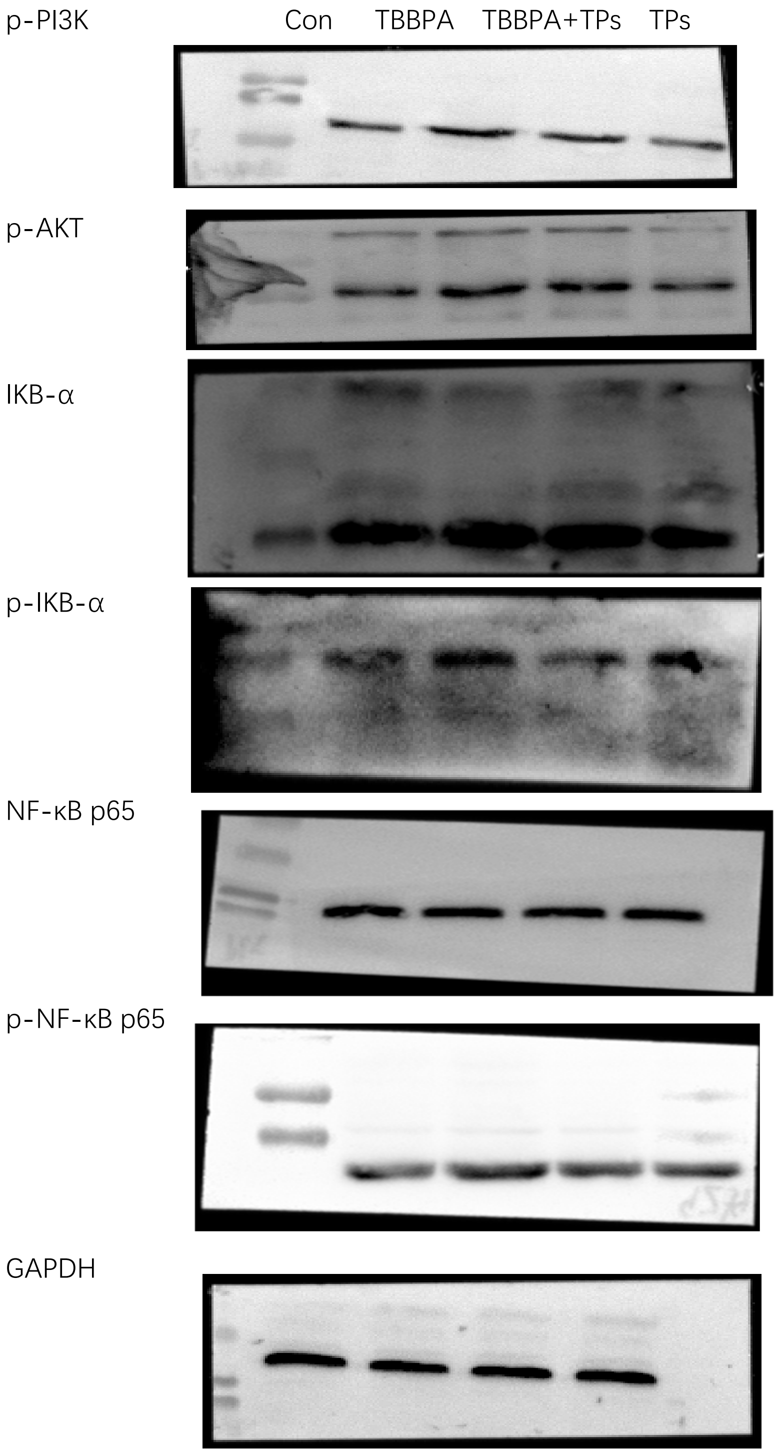
Con
TBBPA
TBBPA+TPs
TPs
p-PI3K
p-AKT
IKB-α
p-IKB-α
NF-κB p65
p-NF-κB p65
GAPDH

BAX

Con TBBPA TBBPA+TPs TPs

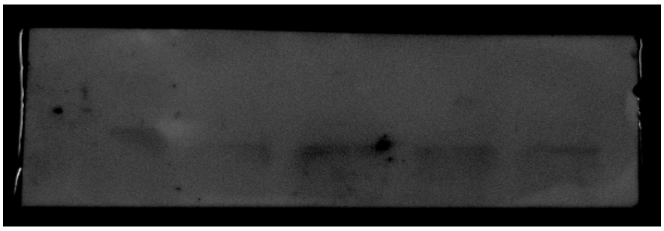

BCL-2

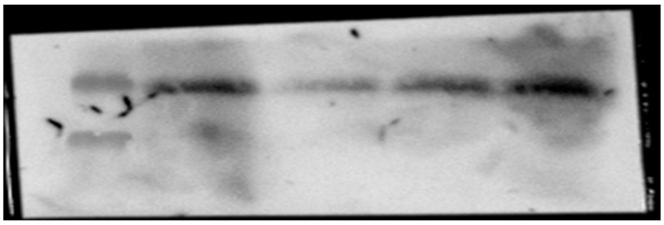

Caspase3

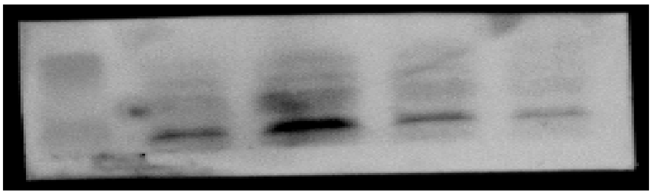

GAPDH

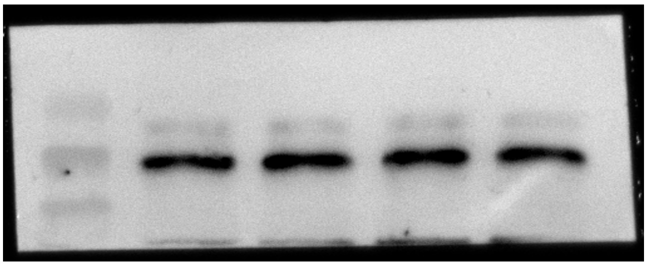

BAX Con TBBPA TBBPA+TPs TPs

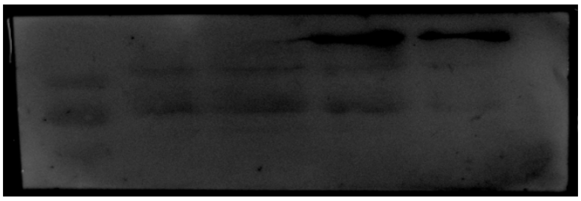

BCL-2

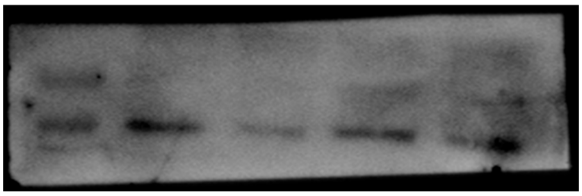

Caspase-3

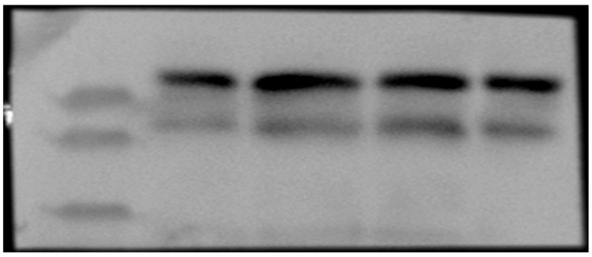

GAPDH

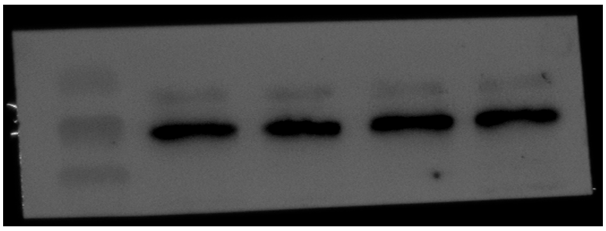

RIPK3 Con TBBPA TBBPA+TPs TPs

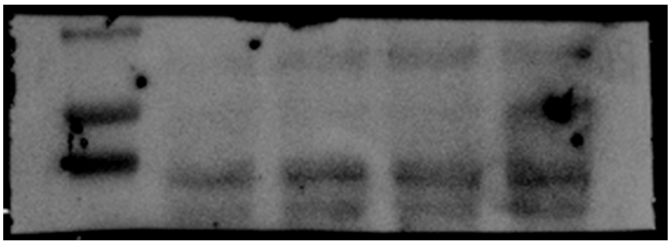

p-RIPK3

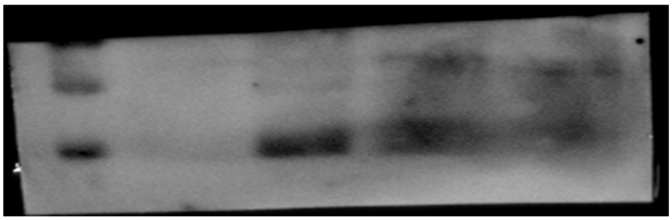

MLKL

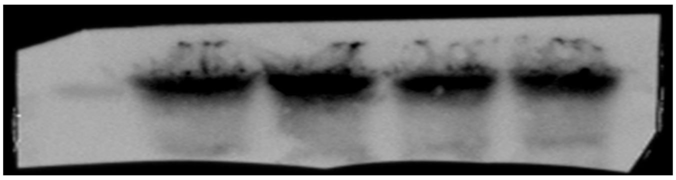

p-MLKL

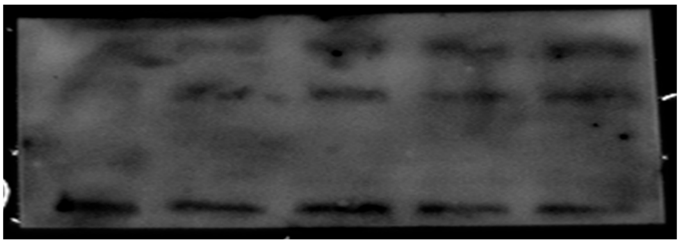

GAPDH

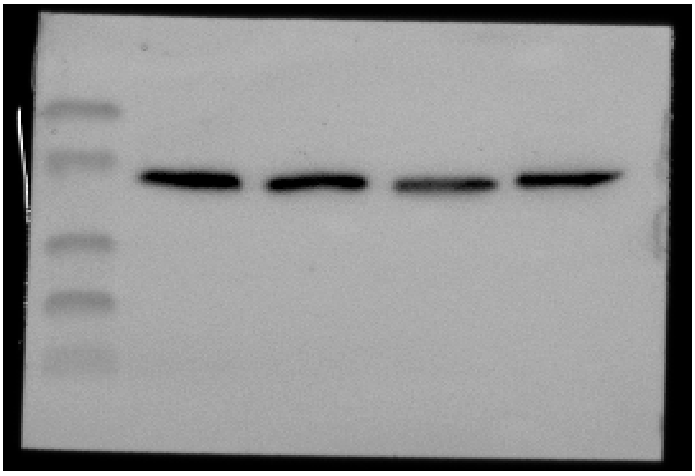

RIPK3 Con TBBPA TBBPA+TPs TPs

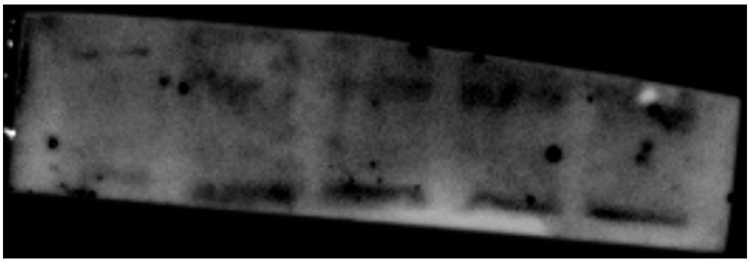

p-RIPK3

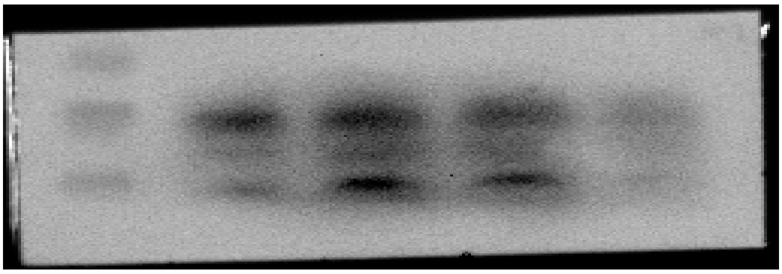

MLKL

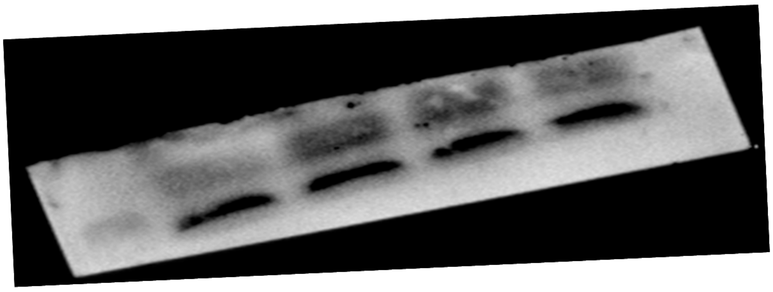

p-MLKL

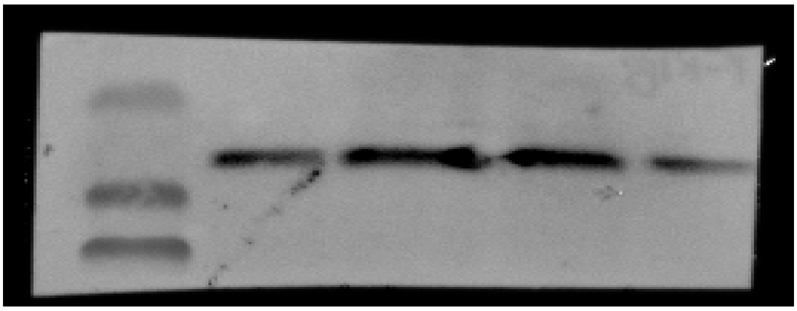

GAPDH

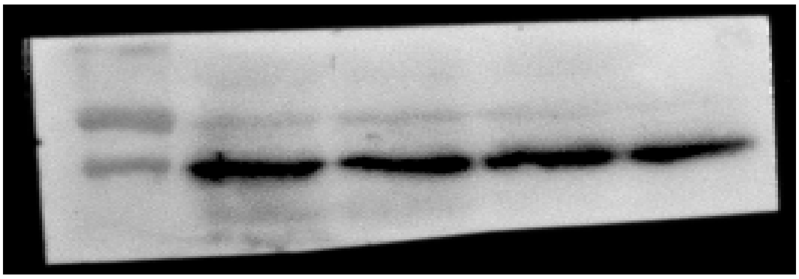

Supplement: Supplementary file 1 [file animals-15-02307-s001.zip › animals-3766723-supplementary.pdf]
